# Epidemiological and Multi-Omics Investigation of Phytosterol Intake and Type 2 Diabetes Risk

**DOI:** 10.21203/rs.3.rs-9222420/v1

**Published:** 2026-04-02

**Authors:** Frank Hu, Fenglei Wang, Anne-Julie Tessier, Andrea Glenn, Yuxi Liu, Marta Guasch-Ferré, Deirdre Tobias, A. Heather Eliassen, JoAnn Manson, Clary Clish, Kyu Ha Lee, Iris Shai, Qibin Qi, Robert Burk, Liming Liang, Qi Sun, Robert Kaplan, Walter Willett, Dong Wang

**Affiliations:** Harvard T. H. Chan School of Public Health; Harvar T.H. Chan School of Public Health; Harvard T. H. Chan School of Public Health; Harvard T. H. Chan School of Public Health; Brigham and Women’s Hospital and Harvard Medical School; University of Copenhagen; Brigham and Women’s Hospital, Harvard Medical School / Harvard TH Chan School of Public Health; Brigham and Women’s Hospital and Harvard Medical School; Department of Medicine, Brigham and Women’s Hospital, Harvard Medical School; Broad Institute of Harvard and MIT; Harvard T. H. Chan School of Public Health; Ben-Gurion University of the Negev; Albert Einstein College of Medicine; Albert Einstein College of Medicine; Harvard T.H. Chan School of Public Health; Harvard T.H. Chan School of Public Health; Departments of Medicine, Epidemiology and Population Health, Albert Einstein College of Medicine; Harvard TH Chan School of Public Health; Harvard Medical School & Brigham and Women’s Hospital

## Abstract

**Objectives:**

Limited evidence exists on the association between dietary phytosterol intake and the risk of type 2 diabetes (T2D). We aimed to investigate this association and identify the underlying plasma metabolic, metabolomic, and gut microbial features.

**Methods:**

We followed 204,633 participants (79% women) from three large US prospective cohorts for up to 36 years. Validated food-frequency questionnaires were used to estimate dietary intake of total phytosterol and three subtypes: β-sitosterol, campesterol, and stigmasterol. We applied Cox proportional hazards models to evaluate their associations with T2D risk. In a subset of 39,879 participants with plasma metabolic biomarkers and 9,528 participants with plasma metabolomics data, we examined the association between phytosterol intake and metabolic biomarkers related to insulinemia, glycemia, lipids, and inflammation, as well as T2D-related metabolomic profiles. Additionally, we explored the gut microbial species and enzymes involved in these associations in a subset of 465 participants with gut microbiome data.

**Results:**

During follow-up, we documented 20,708 incident T2D cases. After adjustment for covariates, higher intake of total phytosterol was associated with a lower T2D risk (HR comparing extreme quintiles = 0.87, 95% CI: 0.82, 0.92; P trend<0.001). Similar associations were observed for β-sitosterol (HR=0.86, 95% CI: 0.81, 0.91; P trend<0.001) and campestrol (HR=0.89, 95% CI: 0.84, 0.94; P trend<0.001), but not for stigmasterol.β-sitosterol and campestrol were also associated with favorable plasma metabolic profiles, such as lower levels of C-reactive protein, leptin, and C-peptide, as well as beneficial T2D-relevant metabolomic profiles. Moreover, we identified several gut microbial species, and their enzymes involved in these associations. For example, *Faecalibacterium prausnitzii* and its β-sitosterol-degrading enzyme 3-oxosteroid 1-dehydrogenase (EC1.3.99.4) were associated with higher β-sitosterol intake and a metabolomic profile indicative of lower T2D risk.

**Conclusions:**

A higher intake of phytosterols, particularly β-sitosterol and campesterol, was associated with a lower risk of T2D, with consistent findings across epidemiological and multi-omics analyses. These findings support the role of a healthy plant-based dietary pattern rich in phytosterol-containing foods such as whole grains, nuts, seeds, fruits, vegetables, and vegetable oils in lowering T2D risk.

## Introduction

Phytosterols are plant-derived steroid compounds naturally present in a variety of plant-based foods, including seeds, cereals, fruits, vegetables, and vegetable oils.^1^ Structurally similar to cholesterol, phytosterols competitively inhibit the intestinal absorption of dietary cholesterol, resulting in reduced blood cholesterol levels, a well-documented property that highlights their potential to improve cardiovascular health.^2–4^ Beyond their cholesterol-lowering properties, animal studies have demonstrated that phytosterols may also exert antidiabetic effects.^5–7^ However, population-based studies evaluating the association between dietary phytosterol intake and the risk of type 2 diabetes (T2D) remain limited. Given the global epidemic of T2D and its substantial public health burden, investigating the role of dietary phytosterols in T2D prevention is of considerable importance.

Recent advances in high-throughput multi-omics technologies, such as metabolomics and metagenomics, offer unprecedented opportunities to unravel the complex molecular profiles underpinning diet-disease associations.^8,9^ Utilizing these approaches, previous studies have identified distinct metabolomic and microbial signatures associated with onset and progression of T2D. For example, alterations in specific metabolites, including branched-chain and aromatic amino acids,^10–12^ as well as shifts in gut microbial communities,^13,14^ have been linked to impaired glucose metabolism, insulin resistance, and chronic inflammation in individuals with T2D. Further investigation of the multi-omics features underlying the phytosterol-T2D relationship may not only enhance our understanding of the epidemiological association but also shed light on the biological pathways through which phytosterols influence glucose regulation and metabolic health.

In this study, we conducted a comprehensive evaluation of the association between dietary phytosterol intake and T2D risk, utilizing data from three large prospective cohorts with up to 36 years of follow-up. In a subset of participants, we further investigated relationships between phytosterol intake and a range of plasma metabolic biomarkers—including measures of inflammation, insulinemia, glycemia, and lipid profiles—as well as metabolomic signatures linked to T2D risk. We also integrated gut microbiome data to identify relevant microbial features. To our knowledge, this is among the first large prospective analyses to link habitual phytosterol intake to incident T2D. We corroborated these findings using clinical metabolic biomarkers and plasma metabolomics data and provided microbiome evidence consistent with phytosterol-related metabolic pathways.

## Methods

### Study design and population

**Figure 1:**
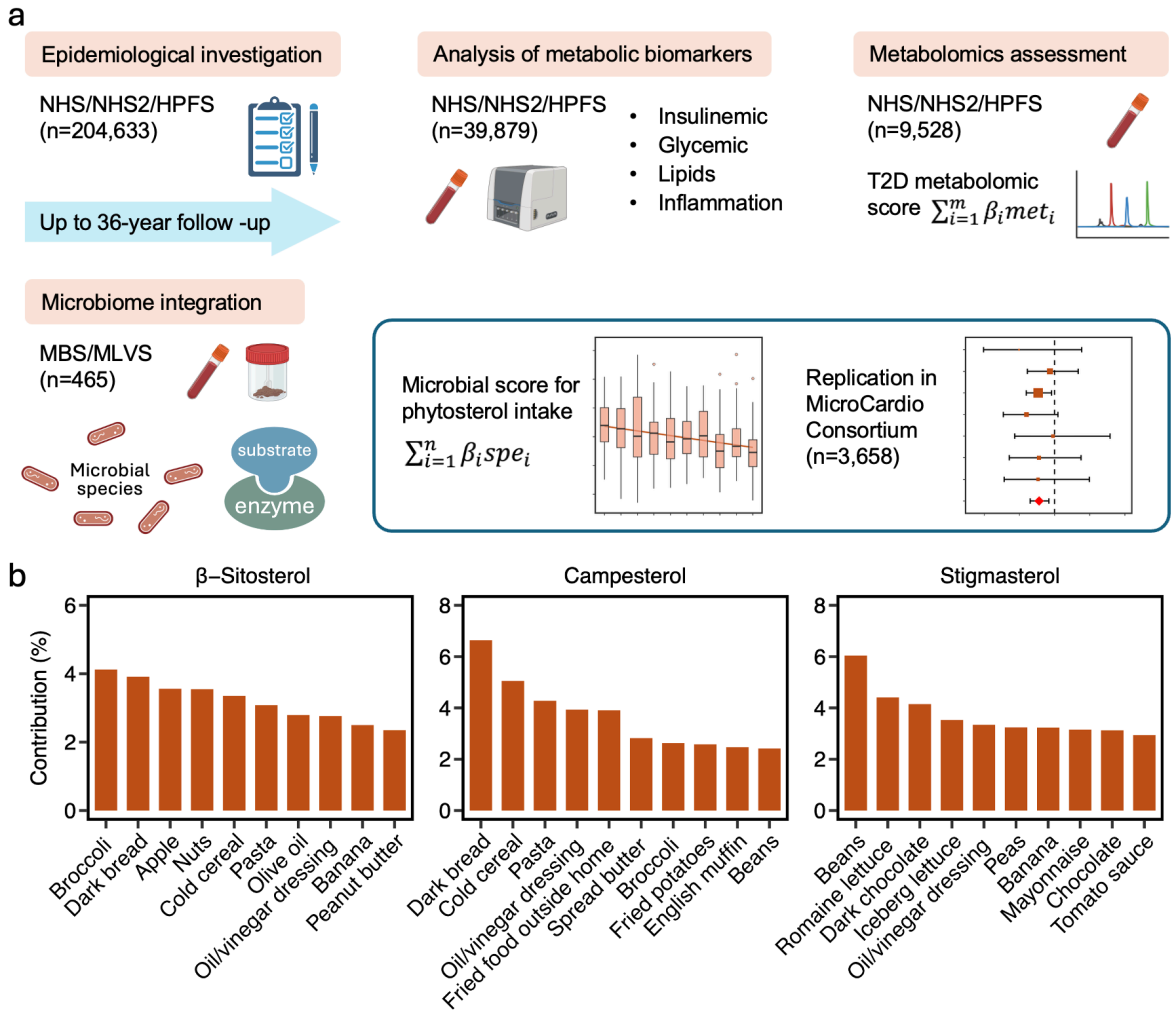
Study Overview. (a). Analytical strategy for the present study. We first examined the association between phytosterol intake and the risk of incident T2D in three large prospective cohorts (n = 206,277). Next, we assessed the relationship between phytosterol intake and plasma metabolic biomarkers in a subset of 39,879 participants, and evaluated metabolomic profiles in a subset of 9,528 participants. Finally, we integrated gut microbiome data to identify relevant microbial features in a subset of 465 participants. We also replicated the association between the phytosterol-related microbial profile and T2D in a large external microbiome consortium (n=3,658). (b).Major food sources of phytosterol intake.

We implemented a multi-cohort, multi-omics study design (Figure 1a). First, we examined the epidemiological associations between phytosterol intake and T2D risk in three prospective cohorts: the Nurses’ Health Study (NHS), NHS2, and the Health Professionals Follow-up Study (HPFS). Among participants from these cohorts who provided blood samples and had metabolic and metabolomic biomarkers measured, we further assessed the relationship between phytosterol intake and these circulating biomarkers. Next, we investigated relevant gut microbial features in two subcohorts with microbiome data: the Men’s Lifestyle Validation Study (MLVS) from HPFS and the Mind Body Study (MBS) from NHS2. Finally, we replicated the association between phytosterol-related microbial profiles and T2D in the MicroCardio consortium. The study protocols for each cohort were reviewed and approved by the Institutional Review Boards of the Harvard T.H. Chan School of Public Health and Brigham and Women’s Hospital.

The NHS, established in 1976, enrolled 121,700 registered female nurses aged 30–55 years, while the NHS2, initiated in 1989, included 116,429 female nurses aged 25–42 years.^15^ The HPFS, which began in 1986, recruited 51,529 male health professionals aged 40–75 years.^16^ Participants across all three cohorts were followed every two to four years using mailed questionnaires to collect detailed information on dietary intake, lifestyle behaviors, and medical histories. In the present study, we excluded individuals who self-reported a diagnosis of diabetes, cardiovascular disease, or cancer at baseline (1984 for NHS, 1991 for NHS2, and 1986 for HPFS). Further exclusions were made for those lacking baseline dietary data or reporting implausible total energy intakes (<500 or >3,500 kcal per day for women and <800 or >4,200 kcal per day for men). Consequently, our epidemiological analyses included 71,660 NHS, 91,602 NHS2, and 43,015 HPFS participants (Figure 1a).

The MLVS included 307 men aged 45 to 80 years who were free from coronary heart disease, stroke, cancer, or major neurological disease.^17^ Between 2011 and 2013, participants provided up to two pairs of self-collected stool samples approximately 6 months apart. The MBS similarly enrolled 233 participants, also free from major diseases, and followed the same protocols as the MLVS, collecting up to two pairs of stool samples from 2013 to 2014.^18^ In both subcohorts, fasting blood samples were collected concurrently with stool samples. The final analysis included 465 participants who had complete data on phytosterol intake, metabolomics, and the gut microbiome (Figure 1a).

The MicroCardio consortium was established in 2021. It is an international collaboration of population-based microbiome studies that integrates both publicly available datasets and those shared via data transfer agreements.^13^ The consortium aggregates individual-level data from participating cohorts, including raw metagenomic sequencing data and extensive phenotypic information such as T2D diagnosis and relevant covariates. For the present study, we included seven cohorts (n=3,658), each of which had a combined number of normoglycemic controls and patients with T2D greater than 100.

### Assessment of dietary intake and covariates

Dietary data for analyses of habitual phytosterol intake and T2D risk was obtained using validated semiquantitative food frequency questionnaires (FFQs)^19,20^ administered every four years—beginning in 1984 and 1986 for the NHS, in 1991 for NHS2, and in 1986 for the HPFS. On each FFQ, participants reported how often, on average, they consumed a standard portion size of each listed food item over the prior year. To calculate the average daily intake of specific phytosterols, the consumption frequency for each relevant food was multiplied by its phytosterol content, and these values were summed across all foods. Nutrient values were estimated based on the Harvard University Food Composition Table, which is largely sourced from US Department of Agriculture data and is supplemented with information from manufacturers and regular updates. The present analysis focused on three primary phytosterol subtypes: β-sitosterol, campesterol, and stigmasterol; total phytosterol intake was determined by summing these three types. The main food sources for each subtype are illustrated in Figure 1b. We also calculated the Alternate Healthy Eating Index (AHEI) to evaluate diet quality.^21^

Information regarding participants’ race, body weight, levels of physical activity, smoking behavior, use of multivitamins, family history of diabetes, and medical histories of hypertension and hypercholesterolemia was gathered from self-reported questionnaires administered every two years. BMI was derived from baseline height and contemporaneous weight data collected during follow-up. Menopausal status and hormone therapy use were also determined among female participants.

### Ascertainment of T2D

Participants who reported a physician diagnosis of diabetes on their biennial questionnaires were sent an additional validated questionnaire requesting further information regarding symptoms, diagnostic testing, and medication use in order to confirm the diagnosis.^22,23^ Only cases of type 2 diabetes (T2D) that fulfilled the National Diabetes Data Group criteria through 1997, or the American Diabetes Association criteria thereafter, were included. Notably, the fasting blood glucose threshold for diagnosis was revised beginning in 1998, with HbA1c subsequently incorporated into the diagnostic guidelines from 2010.

### Measurement of metabolic biomarkers

Blood samples were collected from subsets of each cohort, comprising 32,826 NHS participants (1989–1990), 29,611 NHS2 participants (1996–1999), and 18,159 HPFS participants (1993–1995). Assessment of plasma clinical biomarkers—including C-peptide, HbA1c, total cholesterol, high-density lipoprotein–cholesterol (HDL-C), low-density lipoprotein–cholesterol (LDL-C), triacylglycerol (TAG), C-reactive protein (CRP), adiponectin, and leptin—was conducted in multiple previous substudies following standardized methods.^24,25^ These data were subsequently combined and adjusted for batch variation using an average batch correction approach.^26^ Participants who had a medical history of diabetes, cardiovascular disease, or cancer at the time of blood sampling were excluded, resulting in 39,879 participants (ranging from 5,538 to 15,777 per biomarker) eligible for the final biomarker analyses (Figure 1a). All eligible participants were disease-free at blood collection.

### Metabolomics measurement

High-throughput liquid chromatography–mass spectrometry was employed at the Broad Institute of MIT and Harvard to perform plasma metabolomic profiling within several nested case–control studies. Comprehensive methodological details are available elsewhere.^27,28^ In brief, polar metabolites were identified via hydrophilic interaction liquid chromatography coupled with positive ionization mass spectrometry, and lipids were assessed using C8 chromatography in conjunction with positive ion mode. Pooled plasma references were included every 20 participant samples to correct for instrument drift and batch variance, while blinded quality control samples were distributed at random among study samples for additional quality assurance.

Out of 470 named metabolites identified, we excluded those with an intraclass correlation coefficient <0.3 across blinded quality control samples (n=10) or with ≥ 25% missing data (n=182), leaving a set of 278 metabolites for subsequent analyses. We log-transformed metabolites with highly skewed distributions (absolute skewness >2),^29^ and all metabolites were standardized to z-scores within each substudy. Missing metabolite data were imputed via the random forest approach, in accordance with previous recommendations for metabolomics datasets.^30^ After excluding those with a history of diabetes, cardiovascular disease or cancer at blood collection, the final analytic dataset for metabolomics consisted of 9,528 participants (Figure 1a). As with the metabolic biomarker analyses, all included individuals were free of major chronic diseases at blood draw.

### Metagenomic sequencing and microbial profiling

Protocols for stool sample collection, *ex situ* preservation, laboratory processing, and paired-end shotgun DNA sequencing are described in our prior publications.^13^ Whole-genome metagenomic data were generated using the Illumina HiSeq platform with paired-end reads. Taxonomic composition was determined with MetaPhlAn 4,^31^ while HUMAnN 3.6 was employed for functional profiling.^32^ To be eligible for downstream analysis, a microbial species was required to be present in at least 10% of samples with a minimum relative abundance of 0.01% and to be detected in both the MBS and MLVS substudies. In total, 339 species were included.

### Statistical analysis

We conducted Cox regression models to assess the association between phytosterol intake and T2D risk. Person-time of follow-up for each participant was calculated from the age they returned the baseline questionnaire until the age at onset of T2D, death, or the end of follow-up (June 2020 in NHS, June 2019 in NHS2, and January 2020 in HPFS)—whichever occurred first. We used the updated cumulative average of dietary intake between baseline and the time of censoring to better reflect habitual diet and reduce random measurement error due to within-person dietary variation. All models were stratified by age and calendar year. Multivariable models were further adjusted for BMI, race, family history of diabetes, menopausal status and postmenopausal hormone use (in NHS/NHS2), multivitamin use, smoking, alcohol, physical activity, baseline histories of hypertension and hypercholesterolemia, and dietary factors including total energy, glycemic load, polyunsaturated/saturated fat ratio, and trans fats. The proportional hazards assumption was tested by including interaction terms between phytosterols, and age and calendar year, and no evidence of violation was observed. We conducted analyses separately for each cohort and combined the results via a fixed-effects meta-analysis.

Within the metabolic biomarker subdataset, we utilized multivariable linear regression models to analyze the association between phytosterol intake and plasma metabolic biomarkers. To improve normality, all biomarker values were natural log-transformed; hence, results are expressed as percentage changes: (exp(β-coefficient) – 1) × 100%. Phytosterol intake was averaged from two FFQs completed near the time of blood draw (1986 and 1990 for NHS; 1995 and 1999 for NHS2; and 1990 and 1994 for HPFS). The multivariable models adjusted for age at blood sampling, sex, case-control status within the substudies, fasting status, BMI, race, menopausal and hormone usage status (for women), multivitamin use, smoking, alcohol intake, physical activity, baseline histories of hypertension and hypercholesterolemia, total energy intake, glycemic load, polyunsaturated/saturated fat ratio, and trans fats. We also ran sensitivity analyses restricted to participants who were selected as controls in the original substudies.

To assess whether phytosterol intake is associated with plasma metabolomic biomarkers of T2D, we first constructed a multi-metabolite profile score predictive of T2D risk in the metabolomic subdataset. This score was developed using elastic net penalized Cox regression implemented in a 1,000-fold cross-validation framework. In each iteration, the model was trained on 999 of the folds and the profile score was calculated for the held-out fold. After all rounds, we obtained scores for every participant. The score was computed as a weighted sum of selected metabolites, where the weights corresponded to the coefficients derived from the elastic net regression. We next validated the associations between the metabolite profile score and T2D risk through multivariable Cox regression. Finally, we examined the association of phytosterol intake with the T2D metabolite profile score using multivariable linear regression.

In the microbiome subdataset, we first calculated the T2D metabolite profile score by applying coefficients from the prior metabolomic analysis, using this score as a proxy for T2D risk. To assess the overall association between microbial species and phytosterol intake, we used random forest models to predict phytosterol intake and then determined the correlation between the predicted and actual intake values. To identify specific species associated with phytosterol intake and the T2D metabolite profile score, we log-transformed the relative abundance of each species and applied multivariable linear mixed models with MaAsLin 2, accounting for repeated microbiome profiling. These models incorporated a random effect for each participant and were adjusted for age, BMI, and intakes of total fiber and total energy. Analyses were first conducted separately within MBS (women) and MLVS (men) and then combined through fixed-effects meta-analysis. Additionally, we performed similar analyses for enzymes encoded by the microbial species associated with phytosterol intake.

To investigate the association between the overall microbial profile related to phytosterol intake and T2D directly, we derived a microbial score reflecting phytosterol intake using elastic net penalized linear regression in the microbiome subdataset. This score was then applied to the MicroCardio consortium, where its association with T2D was assessed using logistic regression.

All statistical analyses were two-sided, with a significance cutoff of 0.05 set for T2D outcomes. For analyses involving metabolic biomarkers, metabolites, and microbial features, P values were adjusted for multiple comparisons by controlling the false discovery rate (FDR) using the Benjamini–Hochberg method. An FDR threshold of 0.05 was applied to metabolic biomarkers and metabolites, whereas an FDR threshold of 0.25—commonly used in microbiome research^33,34^—was adopted for microbial features due to the smaller sample size. All analyses were conducted using SAS 9.4 (SAS Institute) and R version 4.2.

## Results

### Characteristics of the participants

Baseline characteristics of participants included in the epidemiological association analyses are presented in **Table 1**. Individuals with higher total phytosterol intake were more likely to be physically active, to use multivitamin supplements, and less likely to smoke. They also tended to consume more healthy plant-based foods—such as whole grains, fruits, vegetables, nuts, and legumes—as well as fish, while consuming less red meat. As expected, phytosterol intake showed moderate positive correlations with the intake of these healthy plant-based foods, the ratio of polyunsaturated to saturated fat intake, total fiber intake, and AHEI, and was inversely correlated with red meat consumption (**Figure S1**).

### Phytosterol intake and T2D risk

During up to 36 years of follow-up, we documented 20,708 incident T2D cases, including 8,667 cases in the NHS, 7,985 in NHS2, and 4,056 in HPFS. Total phytosterol intake was inversely associated with T2D risk. After adjusting for BMI and other lifestyle factors, HR comparing the highest with the lowest quintiles of intake was 0.87 (95% CI: 0.82, 0.92; P trend<0.001). Among the three major phytosterols, only higher intakes of β-sitosterol and campesterol—not stigmasterol—were significantly associated with a lower risk of T2D. The multivariable-adjusted HR comparing the extreme intake quintiles was 0.86 (95% CI: 0.81, 0.91; P trend<0.001) for β-sitosterol and 0.89 (95% CI: 0.84, 0.94; P trend<0.001) for campesterol. These results were generally consistent across the three cohorts (**Table S1**). Restricted cubic spline analyses suggested a linear relationship between total phytosterol and β-sitosterol intake and T2D risk (P for linearity<0.001 for both) (**Figure S2**). However, a slight nonlinearity was observed for campesterol, with the association flattening at higher intake levels (P for nonlinearity=0.02). In sensitivity analyses further adjusting for total fiber intake, the associations for total phytosterol, β-sitosterol, and campesterol were attenuated but remained statistically significant (**Table S2**).

### Phytosterol intake and plasma metabolic biomarkers

**Figure 2:**
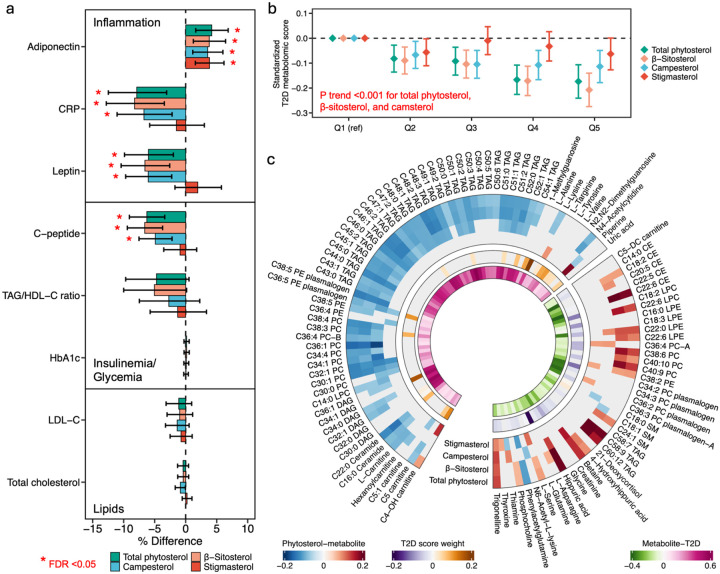
Association between phytosterol intake and plasma metabolic and metabolomic biomarkers for T2D. **(a).** Percentage of differences in eight metabolic biomarkers when comparing the 90th to 10th percentile of phytosterol intake, adjusting for age at blood draw, sex, case-control status, fasting status, body mass index, race, menopausal status and postmenopausal hormone use (NHS and NHS2 only), multivitamin use, smoking status, alcohol drinking, physical activity, baseline history of hypertension, baseline history of hypercholesterolemia, intakes of total energy and trans fat, ratio of polyunsaturated to saturated fat intake, and glycemic load. **(b).** Standardized T2D metabolomics score according to quintiles of phytosterol intake, with the same covariate adjustment as in (a). **(c).** Association between phytosterol intake and T2D-related metabolites. Only metabolites associated with both phytosterol intake and T2D at FDR<0.05, or metabolites selected in the T2D metabolomic score (present in at least 800 out of 1,000 iterations) were shown.

In analyses of plasma metabolic biomarkers, higher intakes of total phytosterol, β-sitosterol, and campesterol were associated with more favorable metabolic profiles (Figure 2a). Specifically, each intake increment corresponding to the difference between the 10th and 90th percentiles, was associated with 3.5–4.2% higher adiponectin, 6.7–8.2% lower CRP, 6.0–6.6% lower leptin, and 4.9–6.6% lower C-peptide levels. Additionally, each increment in stigmasterol intake was associated with 3.8% (95% CI: 1.6, 6.2) higher adiponectin. These results remained largely consistent when analyses were restricted to participants who had been selected as controls in previous substudies (**Figure S3**).

### Phytosterol intake and T2D-related plasma metabolomic profiles

As expected, a higher T2D metabolomic score was significantly associated with higher T2D risk, with an HR of 2.32 (95% CI: 2.14, 2.51) per one standard deviation increment (**Figure S4**). Further analysis assessing the association between phytosterol intake and the metabolomic score indicated that greater intakes of total phytosterol, β-sitosterol, and campesterol were associated with a lower T2D metabolomic score (Figure 2b). The differences in the standardized metabolomic score between extreme intake quintiles were −0.17 (95% CI: −0.24, −0.11; P trend<0.001) for total phytosterol, −0.21 (95% CI: −0.27, −0.14; P trend<0.001) for β-sitosterol, and −0.11 (95% CI: −0.18, −0.05; P trend<0.001) for campesterol, while no significant association was found for stigmasterol. When evaluating the associations between phytosterol intake and individual T2D-related metabolites, higher intakes of total phytosterol, β-sitosterol, and campesterol were generally associated with higher levels of metabolites linked to lower T2D risk, such as trigonelline, betaine, hippuric acid, 4-hydroxyhippuric acid, phosphocholines, and several highly unsaturated lipid subclasses. Conversely, higher phytosterol intake was associated with lower levels of metabolites lined to higher T2D risk, including C22:0 ceramide, L-carnitine, C5 carnitine, and phospholipids and triacylglycerols with low unsaturation (Figure 2c and **Table S3**).

### Gut microbial features linking phytosterol intake and T2D

**Figure 3:**
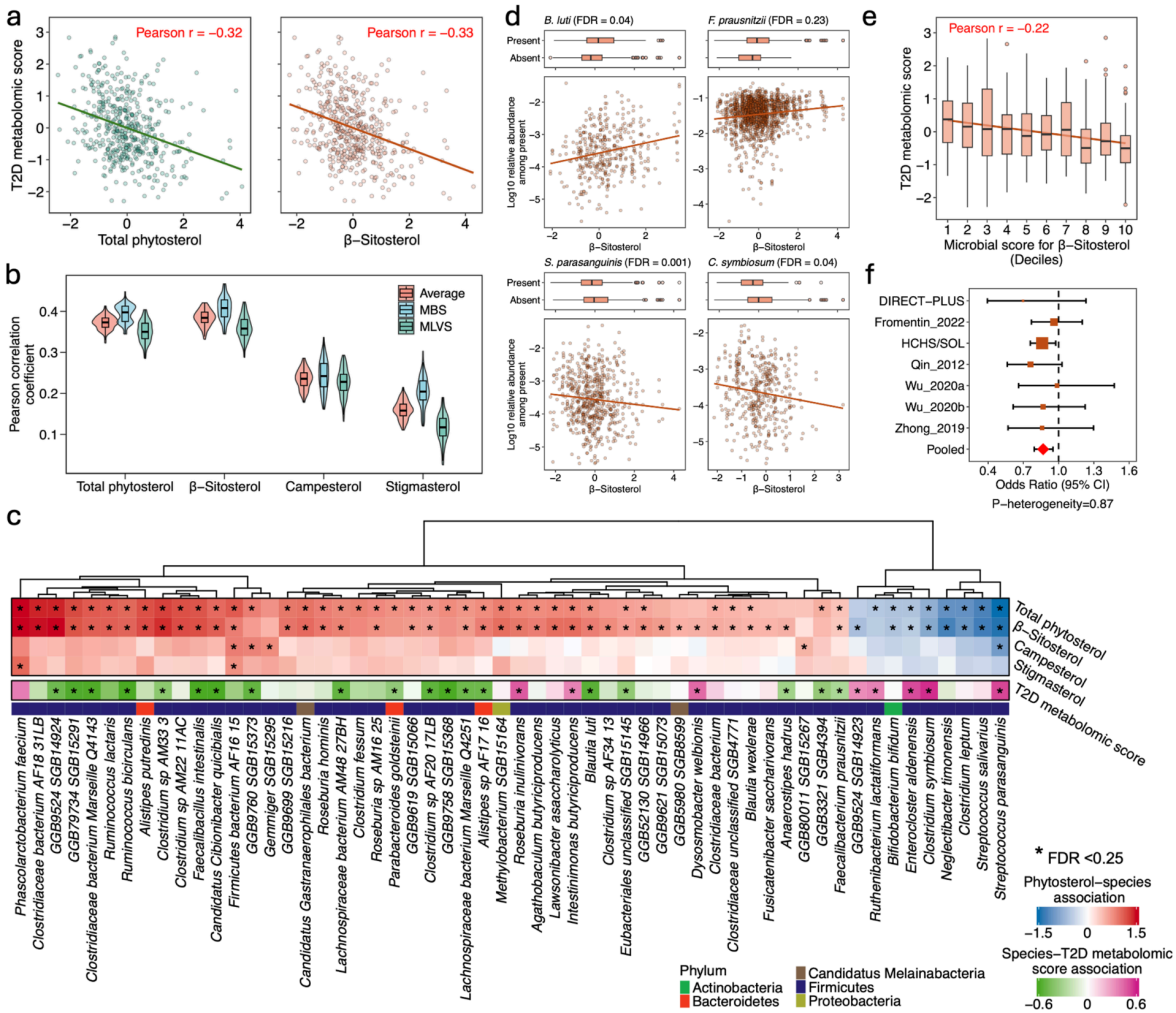
Phytosterol intake and gut microbial composition in relation to T2D. (a). Correlation between intake of total phytosterol and β-sitosterol and T2D metabolomic score in the microbiome subdataset. (b).Correlation between microbial species-predicted phytosterol intake and actual intake. The predicted intake was derived using random forest models. (c).Association between phytosterol intake, microbial species, and T2D metabolomic score. The microbiome analyses were conducted within each study, adjusting for age, body mass index, and intakes of total fiber and total energy, then pooled using a fixed-effect meta-analysis. (d). Representative dose-response associations between phytosterol intake and microbial species. (e). Correlation between a microbial score for β-sitosterol intake and T2D metabolomic score within the microbiome subdataset. (f). Odds ratio of T2D per 1-SD increment of the microbial score for β-sitosterol intake in the MicroCardio consortium. Odds ratios were adjusted for age, body mass index, sex, and metformin use, consistent with other consortium papers.

Using the T2D metabolomic score as a surrogate for T2D risk in the gut microbiome subdataset, we found that higher intakes of total phytosterol, β-sitosterol, and campesterol showed stronger inverse correlation with the score compared to stigmasterol (Figure 3a and **Figure S5**). In examining the relationship between phytosterol intake and overall gut microbiome composition, β-sitosterol showed a stronger correlation between microbial species-predicted and actual intake values compared to campesterol and stigmasterol (Figure 3b). Analysis of individual microbial species identified 47 species associated with total phytosterol intake at FDR<0.25 (Figure 3c and **Table S4**). Further subtype-specific analysis indicated that these species were predominantly associated with β-sitosterol. Species enriched in individuals with higher β-sitosterol intake included *Ruminococcus lactaris*, *R. bicirculans*, *Faecalibacterium intestinalis*, *F. prausnitzii*, and *Blautia luti*, among others, whereas species such as *Streptococcus parasanguinis*, *S. salivarius*, *Clostridium leptum*, *C. symbiosum*, *Enterocloster aldenensis*, and several others were associated with lower β-sitosterol intake (Figure 3d and **Table S4**). Additionally, we assessed associations between phytosterol intake-related species and the T2D metabolomic score. Notably, species associated with higher β-sitosterol intake—such as *F*. *intestinalis*, *F. prausnitzii*, *B. luti*, and *R. bicirculans*—were inversely associated with the T2D metabolomic score, while species associated with lower β-sitosterol intake—including S. *parasanguinis*, *E. aldenensis*, and *C*. *symbiosum*—were positively associated with the score (Figure 3c, **Figure S6**, and **Table S4**).

**Figure 4:**
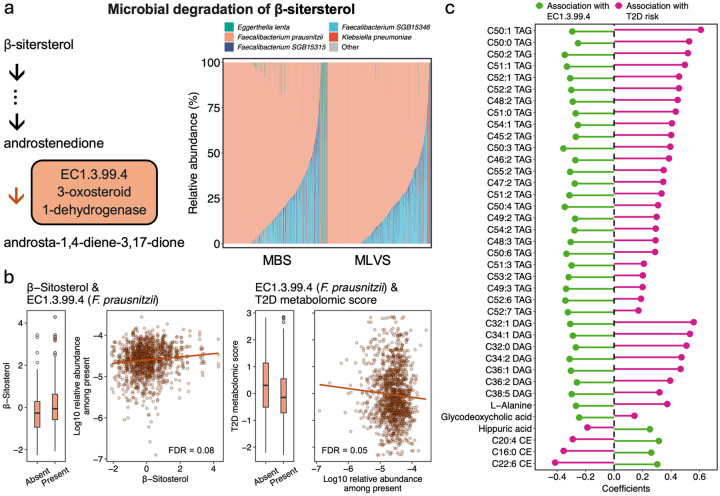
β-sitosterol intake and its microbial degradation in relation to T2D metabolomic profile. (a). Abundance of 3-oxosteroid 1-dehydrogenase by contributing species and samples. (b). Correlation between β-sitosterol, 3-oxosteroid 1-dehydrogenase encoded by *F. prausnitzii*, and T2D metabolomic score. (c). Association of metabolites with 3-oxosteroid 1-dehydrogenase and T2D risk. Only metabolites associated with both 3-oxosteroid 1-dehydrogenase and T2D risk at FDR<0.05 level were presented. Estimates for T2D risk were obtained from the metabolomic analysis in Figure 2c.

In the analysis of enzymes encoded by phytosterol intake-related species, we identified one enzyme involved in the microbial degradation of β-sitosterol—EC1.3.99.4: 3-oxosteroid 1-dehydrogenase—which was predominantly carried by *F. prausnitzii* (Figure 4a). This enzyme was associated with higher β-sitosterol intake and a lower T2D metabolomic score (Figure 4b and **Table S5**). Further analysis of individual metabolites revealed that this enzyme was inversely associated with a series of low-unsaturation triacylglycerols and diacylglycerols, which are in turn linked to a higher T2D risk (Figure 4c and **Table S6**).

In the analysis of the microbial score for β-sitosterol intake, the score was positively associated with β-sitosterol intake (Pearson r = 0.37; **Figure S7**) and inversely associated with the T2D metabolomic score (Pearson r = −0.22; Figure 3e) within the microbiome subdataset. When this score was applied to the MicroCardio consortium, we observed that a higher microbial score for β-sitosterol intake was associated with lower odds of developing T2D (Figure 3f). For each standard deviation increment in the microbial score, the odds ratio of T2D was 0.87 (95% CI: 0.79, 0.95).

## Discussion

Adopting an integrative, population-based approach, we found that higher phytosterol intake—particularly β-sitosterol and campesterol intake—was associated with a lower risk of T2D, independent of BMI and other risk factors. Higher intakes of β-sitosterol and campesterol were also linked to more favorable plasma metabolic profiles and a metabolomic profile indicative of lower T2D risk. Additionally, β-sitosterol intake exhibited the strongest associations with the gut microbiome compared to the other two phytosterol subtypes. We identified several microbial species, as well as a key enzyme involved in the microbial degradation of β-sitosterol, that were associated with both β-sitosterol intake and the T2D metabolomic profile. Moreover, the microbial profile predictive of β-sitosterol intake was associated with lower odds of developing T2D in an external large-scale microbiome consortium.

Given the widely recognized cholesterol-lowering effects of phytosterols, prior research has primarily examined their dietary intake in relation to cardiovascular disease and provided further support for their cardioprotective benefits.^4,35,36^ However, research on the antidiabetic effects of phytosterol has been largely restricted to animal studies,^5–7^ with limited evidence from human populations. Comprehensive population-based studies investigating the associations between phytosterol intake and T2D are scarce. To date, only one prospective cohort study has explored this relationship in the context of the portfolio diet—of which plant sterols are a key component—and reported a beneficial association.^37^ Our study was designed to address this gap, and we observed a lower risk of T2D associated with higher intakes of total phytosterol, β-sitosterol, and campesterol. Our findings provide novel epidemiological evidence supporting the potential antidiabetic benefits of phytosterols in humans and highlight their broader role in metabolic health beyond cholesterol lowering.

Our analyses of plasma metabolic and metabolomic profiles provided biological insights into the beneficial associations of total phytosterol, β-sitosterol, and campesterol intake. Lower C-peptide levels—an indicator of insulin production—observed with higher phytosterol intake suggested a reduced potential for insulinemia. Consistent with this, treatment with β-sitosterol has been shown to restore altered insulin levels in diabetic rats.^6^ Moreover, clinical trials have demonstrated that phytosterol-enriched margarine spread can reduce insulin levels in pregnant women with gestational diabetes.^38^ Additionally, we found that higher phytosterol intake was associated with higher levels of the anti-inflammatory adipocytokine adiponectin, as well as lower levels of proinflammatory metabolic biomarkers such as CRP and leptin. The anti-inflammatory effects of phytosterols have been demonstrated in both in vitro studies and experimental animal models.^39^ It is worth noting that we did not observe a significant association between phytosterol intake and LDL-C, likely because the dietary intake level (<400 mg per day) was substantially lower than the effective daily dose range (1–3 g) for dyslipidemia.^40^ Extending beyond metabolic biomarkers, we integrated metabolomics data to evaluate associations between phytosterol intake and T2D-related metabolomic profiles. We observed that higher intakes of total phytosterol, β-sitosterol, and campesterol were associated with a metabolomic profile indicative of lower T2D risk. Several phytosterol-related metabolites, including trigonelline, betaine, and hippuric acid, can be derived from phytosterol-rich foods—primarily healthy plant foods.^41^ These metabolites may serve as markers of healthy plant-based diets and are associated with improved metabolic health and a lower risk of T2D.^28,42^ Other metabolites—such as C22:0 ceramide, C5 carnitine, and triacylglycerols with low unsaturation—showed inverse associations with phytosterol intake and have been reported to be linked to higher T2D risk.^43–45^ However, further research is needed to elucidate how phytosterols influence these metabolites.

Among the three phytosterol subtypes, we found that β-sitosterol intake was more likely to be associated with gut microbiome composition. This finding is consistent with the lower intestinal absorption rate of β-sitosterol compared to the other two subtypes,^46^ resulting in a greater proportion of β-sitosterol reaching the colon, where it can be acted upon by the microbiota. Research assessing the influence of phytosterol intake on the gut microbiota is scarce. However, the enzyme we identified as being associated with β-sitosterol intake—EC1.3.99.4: 3-oxosteroid 1-dehydrogenase, primarily encoded by *F. prausnitzii*—has been reported in previous studies.^47,48^ This enzyme plays a pivotal role in phytosterol biodegradation, catalyzing the conversion of androstenedione to androst-1,4-diene-3,17-dione, a precursor of sex hormones.^49,50^ Sex hormones are key regulators of lipid metabolism.^51,52^ Our further analysis indicated that this enzyme was inversely associated with low-unsaturation triacylglycerols and diacylglycerols, which are themselves associated with a higher risk of T2D. These findings suggest a potential link between microbial phytosterol metabolism, circulating lipid profiles, and the risk of T2D, highlighting a novel pathway for future investigation.

The main strength of our study is its comprehensive evaluation of dietary phytosterol intake and T2D, utilizing multi-dimensional data in several large prospective cohorts encompassing epidemiological, plasma metabolic and metabolomic, and gut microbiome measures. Such an integrative approach is highly novel and strengthens our findings by providing converging results from different measures that corroborate each another. It also provides potential biological insights that go beyond the observed epidemiological associations. Additional strengths included the long follow-up period, prospective design and repeated dietary assessments for the epidemiological analyses. Nevertheless, our study has several limitations. First, due to the observational design of our study, residual and unmeasured confounding is possible, and causality cannot be established. However, conducting interventional trials to mimic real-word setting and determining the long-term effects of phytosterol intake on T2D risk would be challenging. Second, our FFQ did not capture information on phytosterol supplementation, and phytosterol intake estimates were limited to the three major subtypes. As a result, we were unable to evaluate the potential additional antidiabetic benefits associated with higher levels of phytosterol intake. Finally, the microbiome analyses were constrained by a relatively small sample size, limiting our ability to assess hard T2D outcomes. However, we were able to replicate the association between the β-sitosterol-related microbial profile and T2D in an external, large microbiome consortium with a diverse population background.

In conclusion, higher intake of phytosterols—especially β-sitosterol and campesterol—was associated with lower T2D risk and more favorable metabolic and metabolomic characteristics, with β-sitosterol intake in particular linked to more beneficial gut microbiome profiles. These findings underscore the potential benefits of dietary phytosterols in preventing T2D and support recommendations to follow healthy plant-based dietary patterns rich in phytosterol-containing foods such as whole grains, nuts, seeds, fruits, vegetables, and vegetable oils.

## Supplementary Material

Supplementary Files

This is a list of supplementary files associated with this preprint. Click to download.

- Tables.docx
- PhytosterolintakeandT2DSupplementaryTables.xlsx
- SupplementaryFigures.docx

## Data Availability

The shotgun metagenomic sequencing data from the Nurses’ Health Study II (NHSII) and Health Professionals Follow-up Study (HPFS) are publicly available at the BIOM-Mass Data Portal (https://biom-mass.org/; project names: HPFS and MBS). Due to the gaining of informed consent from the participants, all of the individual-level phenotype data from NHSII and HPFS are available via a request for external collaboration and upon approval of a letter of intent and a research proposal. Details on how to request external collaboration with NHSII and HPFS can be found at https://nurseshealthstudy.org/researchers (contact principal investigator: A. H. Eliassen, email: nhahe@channing.harvard.edu) and https://sites.sph.harvard.edu/hpfs/for-collaborators/ (contact principal investigator L. Mucci, email: lmucci@hsph.harvard.edu).

## Code availability:

Analysis codes are publicly available via https://github.com/fengleiwang/phyto_t2d.
